# Impact of Sodium Glucose Cotransporter 2 Inhibitors (SGLT2i) Therapy on Dementia and Cognitive Decline

**DOI:** 10.3390/biomedicines12081750

**Published:** 2024-08-03

**Authors:** Antonio Lardaro, Ludovica Quarta, Stefania Pagnotta, Giorgio Sodero, Sandro Mariani, Maria Del Ben, Giovambattista Desideri, Evaristo Ettorre, Francesco Baratta

**Affiliations:** 1 Department of Clinical Internal, Anesthesiologic and Cardiovascular Sciences, Sapienza University of Rome, 00161 Rome, Italy; antonio.lardaro@uniroma1.it (A.L.); ludovica.quarta@uniroma1.it (L.Q.); stefania.pagnotta@uniroma1.it (S.P.); maria.delben@uniroma1.it (M.D.B.); giovambattista.desideri@uniroma1.it (G.D.); evaristo.ettorre@uniroma1.it (E.E.); 2 Department of Woman and Child Health and Public Health, Fondazione Policlinico Universitario A. Gemelli IRCCS, 00136 Rome, Italy; 3 Department of Internal Medicine and Medical Specialties, Policlinico Umberto I University Hospital, 00161 Rome, Italy; sandro.mariani557@gmail.com

**Keywords:** sodium glucose cotransporter 2 inhibitors, dementia, mild cognitive impairment, Alzheimer’s disease

## Abstract

Dementia is an age-related syndrome characterized by the progressive deterioration of cognition and capacity for independent living. Diabetes is often associated with cognitive decline and shares similar pathophysiological mechanisms with dementia, such as systemic inflammation, oxidative stress, insulin resistance, and advanced glycation end-products formation. Therefore, adequate diabetes management may reduce the risk of cognitive decline, especially in patients with other comorbidities and risk factors. The sodium glucose cotransporter inhibitors (SGLT2i) regulate renal glucose reabsorption by blocking the SGLT2 cotransporters located in the proximal tubules, causing glycosuria and intraglomerular pressure reduction. Their use helps to lower blood pressure by modifying sodium and water homeostasis; these drugs are also commonly used in the treatment of heart failure and chronic kidney disease, while recently, a potential neuroprotective role in the central nervous system has been suggested. The aim of our scoping review is to analyze current evidence about the potential neuroprotective effects of SGLT2i in adult patients. We performed a scoping literature review to evaluate the effect of SGLT2i on dementia, mild cognitive impairment (MCI) and Alzheimer’s disease incidence and progression. The screening process was performed through different searches on PubMed and EMBASE, evaluating original works published up to January 2024. In conclusion, the use of SGLT2i could be associated with a neuroprotective effect in patients with diabetes, reducing the incidence or the progression of MCI and dementia. Further prospective studies are needed to validate this hypothesis and to evaluate the effectiveness of this class of drugs in normal glycemic profile patients.

## 1. Introduction

Dementia is an age-related syndrome, and it has become a major health burden worldwide in recent decades; this condition is characterized by the progressive deterioration of cognition and capacity for independent living [1]. Data from the World Alzheimer Report 2023 [2] estimates that the population aged over 60 years was 1 billion in 2020, and it is projected to double to 2.1 billion by 2050; approximately 55 million people worldwide are currently affected by dementia and other types of cognitive decline, and this number is expected to rise to 139 million by 2050. Due to the significant impact on patients’ quality of life and the enormous costs for the healthcare system, it is crucial to take action to ensure these individuals can live as healthily and as long as possible [1,2].

There are several risk factors for developing dementia, including various medical conditions, like hypertension or diabetes mellitus (DM); in particular, DM, whether isolated or associated with other clinical conditions, can cause cognitive deterioration, directly through the neurotoxic effect generated by poor glycemic control and indirectly by accelerating the process of cerebral atrophy caused by vascular dementia [3]. In fact, DM shares similar pathophysiological mechanisms with dementia, such as systemic inflammation, oxidative stress, insulin resistance and advanced glycation end-products formation [4,5]. Although the relationship between DM and cognitive decline is largely attributable to metabolic dysregulation and, subsequently, to vascular dementia [6], numerous epidemiological studies have demonstrated that diabetic patients have a higher risk of developing dementia [7]. In fact, insulin resistance could promote β-amyloid (Aβ) plaque accumulation and aberrant tau phosphorylation, which are crucial in the dementia pathophysiological process [8].

Much evidence suggests that adequate DM management may reduce the risk of cognitive decline, especially in patients with other comorbidities and risk factors [6]. In addition to classic medications, like metformin and insulin, there are several more recent glucose-lowering drugs (GLDs) [9], such as glucagon-like peptide-1 receptor agonists (GLP-1RAs), dipeptidyl peptidase 4 (DPP-4) inhibitors and sodium-glucose cotransporter-2 inhibitors (SGLT2i), that improve glycemic homeostasis with a low/absent risk of hypoglycemia.

In particular, SGLT2i, also known as gliflozins, inhibit renal glucose reabsorption by blocking the SGLT2 cotransporters located in the proximal tubules; this mechanism induces glycosuria, intraglomerular pressure reduction and better blood pressure control through a negative sodium and water balance [10]. In addition, SGLT2i have been shown to have a role in reducing progression and morbidity in patients with heart failure and chronic kidney disease and also in non-diabetic patients, independently of their effects on glycemic homeostasis [11,12]. Moreover, there is growing evidence on a potential neuroprotective role of SGLT2i in various type of neurodegenerative cognitive decline. SGLT receptors are expressed in brain regions involved in the learning process and food intake regulation, and SGLT2i exert neurological beneficial effects by which gliflozins might improve cognitive function in subjects with diabetes mellitus and Alzheimer’s disease (AD) [13]. However, there are no univocal data on the effects of this drugs on the risk of dementia or its incidence and progression [14].

The aim of this review is to summarize the current evidence on the association between SGLT2i use and dementia, mild cognitive impairment and Alzheimer’s disease incidence and natural history.

## 2. Materials and Methods

### 2.1. Study Selection

**Figure 1:**
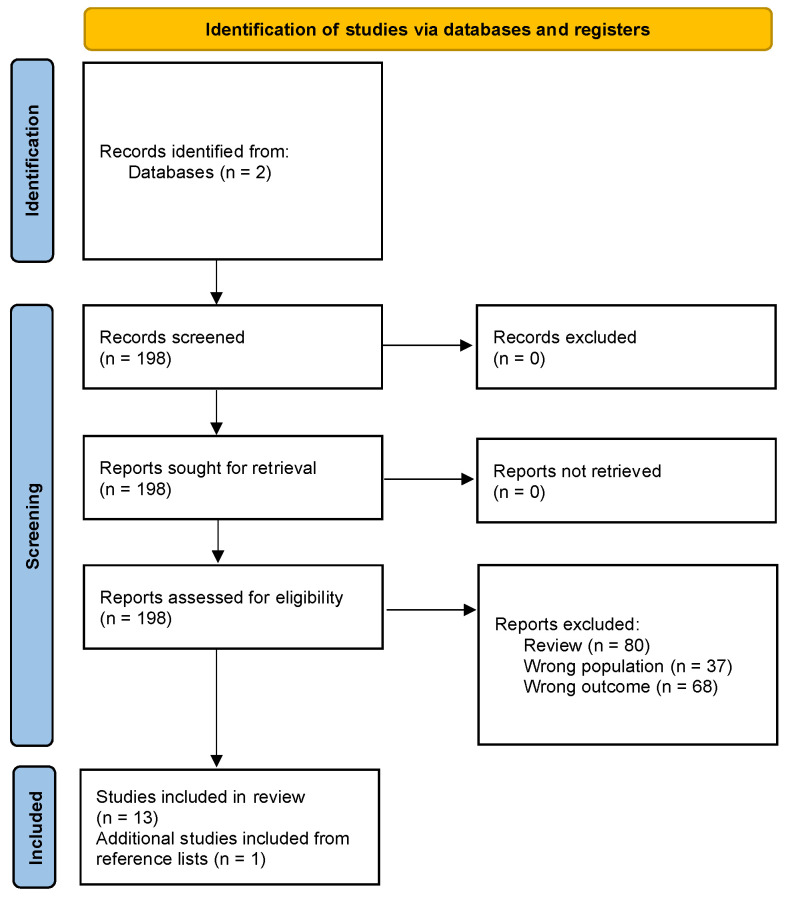
Scoping review flow diagram according to the PRISMA 2020 statement.

Our scoping review was performed with the Preferred Reporting Items for Systematic Reviews (PRISMA) extension for scoping reviews. The literature search strategy was aimed at identifying clinical studies evaluating the association between SGLT2i therapy and dementia incidence and natural history; we considered all type of cognitive decline, including mild cognitive impairment. The screening process (Figure 1) was performed through different searches on PubMed and EMBASE. Our search string combined the following keywords: ((((sglt2) OR (sglt2i)) OR (sglt2 inhibitors)) OR (dapagliflozin)) AND ((((((Alzheimer) OR (MCI)) OR (dementia)) OR (mild cognitive impairment)) OR (cognitive)) OR (neuroprotective)). We considered eligible for our review all original works published up to January 2024.

Our scoping search was conducted according to the following SPIDER approach: Sample—adult patients diagnosed with dementia or at risk of cognitive decline; Phenomenon of Interest—therapy with SGLT2 inhibitors (SGLT2i); Design—randomized clinical trials, longitudinal studies and cross-sectional studies; Evaluation—association between SGLT2i use and the incidence and natural history of dementia, mild cognitive impairment or Alzheimer’s disease; Research type—qualitative or quantitative methods. Articles not fully available and not in English were excluded from the preselection process. In order to identify research about the possible neuroprotective effect of SGLT2i, two physicians (A.L. and L.Q.) performed an initial selection of original articles based on abstracts. A third physician (F.B.) reviewed the eligible studies for appropriateness and completeness. Disagreements on study selection and data extraction were resolved by consensus among the authors.

From the initial studies selection (n = 198), reviews articles (n = 75 reviews; n = 5 systematic reviews) and off-topic studies (n = 105; n = 37 conducted on animals and n = 68 with a different primary outcome) were excluded. A total of 13 publications were fully considered and included in this review. Additionally, we analyzed the reference lists of the included articles and excluded reviews to identify further relevant studies (n = 1). A total of 14 studies were included in the review. More details regarding our article screening and selection process are reported in the PRISMA diagram (Figure 1).

### 2.2. Data Extraction and Synthesis

The summarized information extracted from the 14 included articles are reported in Table 1.

The data extraction was conducted by two authors (A.L., L.Q.) filling a pre-defined Microsoft Office Excel form, reporting references with the DOI, authors, authors’ country, year of the study, type of study, main aim and secondary aims (if reported), number of patients, type of patients, information about diabetes, age in every group, follow-up period, type of SGLT2i used, side effects (if observed) during treatment and main results. If the authors were in disagreement, a third one (S.P.) was consulted. We also analyzed information about limitations and any eventual conflicts of interest among the authors, if reported. Due to the heterogeneity of the publications analyzed, it was not possible to perform a meta-analysis of the results.

## 3. Results

After our screening process, we fully included 14 publications [15,16,17,18,19,20,21,22,23,24,25,26,27,28] in our scoping review, with a total of 590,017 analyzed patients. All studies recruited diabetic patients; therefore, no information about the benefits in patients without glycemic disorders is available (Table 1).

Most of the studies were conducted on patients without dementia at baseline (seven studies) [16,17,19,21,23,24,26]. Four studies analyzed patients with and without cognitive alteration [15,18,27,28], another publication only analyzed patients with mild cognitive impairment (MCI) [21], only one study enrolled patients with dementia [22] and the last one analyzed both patients with MCI and patients with dementia [24].

In none of the studies was a distinction between the various types of dementia made. Therefore, patients with AD (which represents the most frequent form of dementia) were considered together with other types of cognitive decline, except for MCI.

The publication year varied from 2018 to 2024; most articles were published in 2022 (n = 6), highlighting the importance of our topic in light of recent scientific evidence. Many publications analyzed patients from Italy and China. We decided to discuss the results separately based on the type of scientific article (randomized clinical trials, longitudinal studies and cross-sectional studies).

### 3.1. Randomized Clinical Trials

Of the 14 articles selected, 3 were randomized clinical trials, conducted in all cases on diabetic patients. In one study [20], the authors recruited patients with a diagnosis of Mild Cognitive Impairment, while in the other two publications [16,19], the authors considered patients with normal cognitive function at the baseline. Overall, the studies included 171 patients (96 with MCI; 75 without a codified diagnosis of cognitive disorder), of which 81 were treated with SGLT2i.

Haiyan Cheng et al. [16] did not find a cognitive benefit of adding dapagliflozin therapy in patients with poorly controlled diabetes with metformin, instead highlighting a short-term cognitive improvement with the addition of liraglutide. Perna et al. [19], considering a more extended follow-up (12 months), found a metabolic benefit of SGLT-2i therapy in the absence of changes in cognitive status, while Zhao Y et al. [20] demonstrated that the addition of dapagliflozin therapy, together with cognitive psychological training, improved patients’ cognitive status, with an increase in MMSE and other indirect indices of mental health.

### 3.2. Longitudinal Studies

In our analysis, we included nine longitudinal studies, with a total of 302,073 diabetic patients and 158,516 SGLT2i users. Juraj Secnika et al. [18] recruited patients with and without cognitive impairment diagnoses. The authors demonstrated an improvement in the survival rate in SGLT2i users with a previous diagnosis of dementia, in contrast to subjects with normal cognitive function. Mone P et al. [24] also conducted a study on 162 patients with a previous diagnosis of dementia and MCI, confirming a short-term (1-month therapy) improvement in the Montreal Cognitive Assessment (MOCA) scores and physical performance in patients treated with empagliflozin.

The remaining five studies [17,21,23,25,26] were conducted on patients with normal cognitive performance at baseline and highlighted a possible cognitive benefit of SGLT2i.

Three of these articles [17,21,25] aimed to assess cognitive function and the incidence of dementia in patients with diabetes, while the other two [23,26] also evaluated the incidence of Parkinson’s disease and the rate of cardiovascular mortality.

Proietti et al. [26] conducted an observational study and examined the effect of SGLT2i therapy on reducing the occurrence and risk of cerebrovascular events, incident dementia, heart failure and mortality in a cohort of patients with atrial fibrillation (AF) and type 2 diabetes mellitus. They reported a higher risk of dementia among patients not receiving SGLT2i therapy (HR 1.66, 95% CI 1.30–2.12; log-rank *p* = 0.001). Additionally, the study found an increased risk of ischemic stroke/transient ischemic attack and intracerebral hemorrhage among patients not receiving SGLT2i compared to those taking SGLT2i. Survival free from incident heart failure was lower in patients using SGLT2i, while the mortality risk was higher among patients not receiving SGLT2i.

Che-Yuan Wu et al. [21] also conducted a comparative analysis of SGLT2i and DPP-4i users, demonstrating that the former had a lower risk of developing dementia. In their subgroup analysis, they further highlighted that among the various SGLT2 inhibitors used, dapagliflozin exhibited the most favorable preventive efficacy profile.

### 3.3. Cross-Sectional Studies

Two cross-sectional studies were included in our review, with a total of 74,647 patients and 1161 SGLT2i users; in both studies, patients with cognitive impairment were recruited. Bohlken J. et al. [15] did not highlight a benefit of SGLT2i therapy in reducing the prevalence of dementia, instead confirming a significant benefit regarding the risk of cognitive deterioration during metformin administration as monotherapy or associated with other antidiabetic drugs. Different results were found by Wium-Andersen et al. [22], who found reduced dementia odds following antidiabetic therapy and a statistically more significant improvement with SGLT2i and aGLP1.

### 3.4. Summary of Results

Six publications analyzed the impact of SGLT2i on dementia incidence and its effects on cognitive performances and anthropometrics parameters; the follow-up period of the studies was not uniform (Bohlken et al. [15], with a follow up period of at least 1 year; Siao et al. [17], with approximately 2.4 years; Wium-Andersen et al. [22], with a medium follow-up time of 7.2 years; Low et al. [25], with 4.6 years; Jing Cheng Ding et al. [27], with between 16.1 and 16.4 months; and Eugene Merzon et al. [28], with 12 months). Most of the analyzed publications (four studies) showed a neurocognitive benefit of SGLT2i. Eugene Merzon et al. [28] highlighted that adults with MoCA < 19 had more diabetes-related complications and were less likely to be treated with antidiabetic drugs, except insulin, while Bohlken et al. [15] observed no cognitive benefit of SGLT2i during a short observational period.

Two studies [19,21] compared the effects of cognitive performances between gliflozin and incretin users; however, only Wu C.Y. demonstrated the superiority of SGLT2 inhibitors over DPP4 inhibitors. One publication [16] concerned the effect of a short treatment with various GLDs (liraglutide, dapagliflozin or acarbose) on odor-induced brain functional alterations and cognitive changes, while two studies (Secnika et al. [18] and Proietti et al. [26]) analyzed all causes of mortality among users of GLDs in dementia and dementia-free subjects.

Zhao Ying et al. [20] investigated the cognitive and metabolic benefits of dapagliflozin in elderly patients with diabetes and mild cognitive impairment, while Mone et al. [24] conducted a similar analysis on patients with diabetes and concomitant heart failure, also evaluating differences with insulin and metformin therapy. Mui et al. [23] compared the incidence of dementia, Parkinson’s disease and cerebrovascular mortality between SGLT2i users and DPP4i users. The side effects of SGLT2i were not investigated in any studies. Merzon et al. [26] evaluated the prevalence, clinical characteristics and healthcare utilization among patients with type 2 diabetes and previously undiagnosed cognitive impairment.

In summary, our scoping review identified few studies investigating the potential neuroprotective effects of SGLT2 inhibitors on the incidence and progression of cognitive decline, revealing conflicting results. In addition, the high variability of outcomes, follow-up durations and study designs did not allow for meta-analyzing the findings. Currently, there is insufficient scientific evidence to recommend their use in patients diagnosed with or at risk of cognitive decline, unless they are prescribed for other clinical conditions such as diabetes mellitus, heart failure or chronic kidney disease [9,10,11]. Nonetheless, the findings from our review provide the groundwork for future trials aimed at evaluating the efficacy of these drugs in Alzheimer’s disease and other forms of cognitive decline. The objective is to enhance the quality of life for these patients and to slow disease progression.

## 4. Discussion

Currently, six SGLT2i (ipragliflozin, dapagliflozin, canagliflozin, empagliflozin, luseogliflozin and tofogliflozin) are available for type 2 diabetes treatment, while in some countries, only two of them (Ipragliflozin and dapagliflozin) have been additionally approved for type 1 diabetes treatment [29]. This class of drugs has recently also been approved for the treatment of heart failure, independently of ejection fraction (preserved, mildly reduced or reduced), because it can reduce morbidity and mortality in these patients [30]; clinical benefits were similarly observed in patients with or without diabetes [31].

The just-mentioned vascular benefit is particularly evident in patients with chronic kidney disease and reduced glomerular filtration [32], with a major reduction in the mortality rate and a possible reduction in hemorrhagic stroke risk in selected categories of patients.

A recent meta-analysis [8] summarized the results of three observational studies included in our review [15,22,23], highlighting how SGLT2 inhibitor use was significantly associated with a decreased risk of all-cause dementia compared to non-SGLT2 inhibitor use (RR, 0.62; 95% CI, 0.39–0.97), despite the high heterogeneity of the articles considered; in the same publication, the authors highlighted a similar benefit for DPP-4i and for GLP-1RAs, emphasizing the benefit related to general dementia, while only partial data are available for the various subtypes.

In addition to the cardiac and kidney effects, SGLT2i could also have antiplatelet and antithrombotic activity related to NOX2 pathway down-regulation [33], which can be synergistic with the use of other drugs, reducing cardiovascular risk [34]

Moreover, in elderly patients, the administration of SGLT2i and the modulation of glucose metabolism also have a protective role in the bone by regulating the turnover of the skeletal microarchitecture [35]. The efficacy in the prevention of fractures is controversial and differs on the basis of the type of drug used: in some studies, empagliflozin and ertugliflozin may increase the risk of fracture, while dapagliflozin may show a potential protective effect [35], especially when combined with other antidiabetic drugs. In this scenario, SGLT2i act in multiple parallel ways in elderly multimorbid patients.

Due to the recent approval and limited indications, the neuroprotective role of SGLT2i in patients without impaired glucose metabolism is under-investigated, and all studies have been conducted on diabetic patients, who have an increased risk of cognitive decline [6]. The results from the clinical studies are heterogenous and do not unequivocally prove a neuro-protective role of SGLT2i in the elderly.

Some evidence showed no beneficial effect of SGLT2i on cognitive performance in patients with T2D. Haiyan Cheng et al. [16] compared the add-on of liraglutide, dapagliflozin or acarbose in patients with poorly controlled diabetes with metformin, highlighting a cognitive improvement only in the liraglutide group. Although the authors did not observe a benefit of using SGLT2i, the data are limited to a relatively short follow-up (only 16 weeks), and studies with more extensive follow-ups could lead to different results. However, Perna et al. [19], conducing a more extended comparison between SGLT2i, DPP-4 inhibitors and GLP-1-RA with 12 months of follow-up, demonstrated that the cognitive test scores and, in general, the cognitive performance did not change in any group; this finding is probably related to euglycemia associated with the attenuation of oxidative stress.

Cheng et al. [16] demonstrated a significant activation of brain olfactory-related regions of the left hippocampus following liraglutide therapy but not after dapagliflozin administration, with a concomitant improvement in delayed memory. The initial stages of cognitive decline could be characterized by a reduction in the functionality of the olfactory areas of the brain [36]; in a study conducted by Marigliano et al. [37], it was demonstrated that the olfactory deficit had a sensitivity of 92.3% in identifying patients with progression to Alzheimer’s disease at one year of follow-up, suggesting a possible utility of olfactory tests for early diagnosis and for identifying patients at risk of progression. Olfactory stimulation is part of dementia treatment and care, improving the quality of life of patients [38]; however, it is not known whether the pharmacological stimulation of brain areas related to smell can provide a benefit regarding the progression of cognitive decline.

The neurocognitive benefit of SGLT2i is shared with other antidiabetic drugs—in particular, with metformin, which is currently the first-line therapy [39] in diabetic patients at risk of developing dementia or Alzheimer’s disease. Jens Bohlken et al. [15] confirmed the benefit of metformin; moreover, they conducted one of the studies included in our review, where a neurocognitive benefit of SGLT2i was not demonstrated, as the reduction in the incidence of dementia was not statistically significant (*p* = 0.477). In the same study, the authors highlighted that insulin therapy, unlike other antidiabetic drugs, was a risk factor for the development of dementia and for equal pathological levels of glycated hemoglobin and other indicators of glycemic unbalance; this result is partially explained by the fact that patients with type 2 diabetes on insulin therapy have a more severe disease, less controlled by first-line drugs, and therefore have a higher risk of comorbidities.

Conversely, a recent meta-analysis [8] summarized the results of three observational studies included in our review [15,22,23], highlighting how SGLT2 inhibitor use was significantly associated with a decreased risk of all-cause dementia compared to non-SGLT2 inhibitor users (RR, 0.62; 95% CI, 0.39–0.97), despite the high heterogeneity of the articles considered; in the same publication, the authors highlighted a similar benefit for DPP-4i and for GLP-1RAs, emphasizing the benefit related to general dementia, while only partial data are available for the various subtypes.

The benefit of the proper pharmacological management of diabetes can also reduce the mortality rate of patients with cognitive decline. Secnik et al. [18] extracted data from diabetic patients from five Swedish national registers, highlighting that the mortality rate in patients with dementia using metformin (the most prescribed antidiabetic in their cohort) was not different from that of non-users, while the use of SGLT2 inhibitors was associated with a 57% lower mortality rate in new users with dementia. The reduction in the mortality rate was not confirmed in patients with normal cognitive function. The lack of significant differences in mortality among patients with a normal cognitive status is an aspect that requires further investigation.

Another study [20] further supported this hypothesis, demonstrating the benefit of SGLT2 inhibitors in the progression of mild cognitive impairment (MCI) and a statistically significant improvement in the Mini-Mental State Examination (MMSE). The benefit is only partially attributable to the antidiabetic therapy, as the selected patients also underwent cognitive training. However, the positive effect of administration is supported by the improvement in blood glucose and glycated hemoglobin levels, which are associated with the risk of vascular dementia.

The SGLT2i neurocognitive protection could result from several neuroprotective effects reported in the literature. Experimental data demonstrated cerebral microvascular circulation improvement after SGLTi administration in DM animal models [13]. Anti-apoptotic, anti-inflammatory and anti-oxidant effects of SGLT2i were reported [33,40,41], with a specific improvement in the mitochondrial function [42] and a consequent positive remodeling of the neurovascular unit [43]. The effects on mitochondrial function might partially depend on the known effects of gliflozin in restoring mTOR dynamic cycling, which is chronically activated in both DM and AD patients [44]. SGLT2i could also exert their neuroprotective effects by mitigating the postischemic hyerglycemia-induced neuronal damage [45]. Moreover, the use of SGLT2i counteracts the aberrant neuronal remodeling consequent to glucotoxicity [43], promotes immature neurons generation and increases synaptic density [41]. Finally, SGLT2i reduce the hyperphosphorylated tau levels and amyloid β (Aβ) accumulation and enhance brain insulin signaling [46,47].

Our scoping review has some limitations. The potential use of SGLT2i therapy for neuroprotective purposes is a recent topic, and it is possible that several clinical studies with interesting results are currently underway but not yet published. The choice to use a single database did not allow for a systematic review of all existing literature in this field. Moreover, it is possible that our keywords did not encompass all the available literature on this topic. In addition, there were no RCT studies investigating this issue, and the wide heterogeneity of the observational studies did not allow for performing a conclusive meta-analysis of the findings.

In summary, few studies investigated SGLT2i’s effect on neurocognitive function, and the results were conflicting. However, this is an interesting scoping review that lays the groundwork for future meta-analyses regarding the neuroprotective action of SGLT2i and potential clinical trials on the subject.

## 5. Conclusions

Pre-clinical studies and some of the observational ones conducted on the neuroprotective role of SGLT2i might indicate a new possible indication for the use of these drugs. SGLT2 inhibitors may play a preventive role in the progression of organic changes associated with the onset and progression of dementia and improve the patient autonomy degree even better than other antidiabetic drugs (e.g., metformin) [24].

Finally, ad hoc RCTs might produce the needed evidence to support the use of SGLT2i to prevent and treat neurocognitive decline in both patients with and without diabetes.

## Figures and Tables

**Table 1:** General characteristics of studies included in our scoping review.

Author	Type of Study	Aim(s)	Patients Number	Age	SGLT2i	Time of Observation	Results
Bohlken J et al. (Germany) [15] (2018)	Cross-sectional study	To analyze the association between the use of antihyperglycemic drugs and dementia risk in patients followed in general practices	8276 patients with dementia vs. 8276 dementia-free patients	N/A	not reported	N/A	Metformin use (in monotherapy or in combination with sulfonylureas) and glitazone use were negatively associated with dementia risk (*p* < 0.001), while insulin use was positively associated with dementia risk. Regarding SGLT2i, there was no significant difference in the risk of developing dementia (*p* = 0.477)
Cheng H. et al. (China) [16] (2022)	Randomized clinical trial	To investigate the therapeutic effects of liraglutide, dapagliflozin or acarbose treatment on brain functional alterations and cognitive changes in patients with type 2 diabetes	12 dapagliflozin users, 12 liraglutide users, 12 acarbose users	57 (9.5 SD) years in the SGLT2i group; 51.9 (10.2 SD) years in the Liraglutide group; 56.4 (8.9 SD) years in the Acarbose group	dapagliflozin	16 weeks	Liraglutide significantly enhanced the impaired odor-induced left hippocampal activation with Gaussian random field correction and improved cognitive subdomains of delayed memory, attention and executive function (all *p* < 0.05), whereas dapagliflozin or acarbose did not
Siao W.Z. et al. (Taiwan) [17] (2022)	Longitudinal Study	To explore the association between SGLT2i and dementia incidence in patients with type 2 diabetes	103,247 SGLT2i users. 103,247 non-SGLT2i users	not reported	canaglifozin, dapaglifozin, empaglifozin.	32 Months	After the adjustment for gender, age and comorbilities, the SGLT2 inhibitor group was associated with a lower risk of incident dementia compared to the non-SGLT2 inhibitor group (aHR: 0.89; [95% CI 0.82–0.96])
Secnik J. et al. (Sweden) [18] (2022)	Longitudinal Study	Analyze all-cause mortality among users of glucose-lowering drugs in dementia and dementia-free subjects	11,401 patients with dementia and 121,001 dementia-free patients	75.7 (6.3) years in the SGLT2-i group; 79.8 (7.1) years in the control group	not reported	13 years	GLP-1a (HR: 0.44 [95% CI 0.25–0.78]) and SGLT-2i users with dementia (HR 0.43 [95% CI 0.23–0.80]) experienced lower mortality compared to non-users
Perna S. et al. (Italy) [19] (2018)	Randomized clinical trial	To examine the effects on cognitive performance, anthropometric measures and metabolic markers in two different treatments: Incretins vs. SGLT2i	18 incretin users vs. 21 SGLT2i users	77.21 (8.07 SD) years.	canaglifozin, dapaglifozin, empaglifozin.	12 months	Cognitive status did not change significantly during the 12 months of treatment in both groups
Zhao Y. et al. (China) [20] (2021)	Randomized clinical trial	To investigate the effect of dapagliflozin combined with cognitive behavior training on quality of life and cognitive function in elderly patients with type 2 diabetes mellitus and mild cognitive impairment	48 patients with standard cares for diabetes vs. 48 patients with dapagliflozin combined with cognitive behavior training	59.4 (8.0 SD) in controls and 61.0 (8.43 SD) in the experimental group	dapaglifozin	1.5 years	Dapagliflozin combined with cognitive behavior training intervention improve cognitive function, the self-efficacy of diabetes management and quality of life
Wu C.Y. et al. (Canada) [21] (2022)	Longitudinal Study	To investigate the association between SGLT2i and DPP-4i and the incidence of dementia.	36,513 SGLT2i users and 70,390 DPP-4i users	72.4 (5.4 SD) years in the SGTL2i group and 74.3 (6.5 SD) years in the DPP-4i group	canaglifozin, dapaglifozin, empaglifozin.	2.80 years	SGLT2i compared with DPP-4i were associated with a lower risk of dementia (14.2/1000 person-years; aHR 0.80 [95% CI 0.71–0.89])
Wium-Andersen I.K. et al. (Denmark) [22] (2019)	Cross-sectional study	To investigate the association between different types of antidiabetic medication and treatment regimens (combinations of antidiabetic drugs) and dementia diagnoses in patients with type 2 diabetes.	176,250 patients	70.8 (51–67 IQR) years in the dementia group and 59.0 years (64–78 IQR) in the dementia-free group	not reported	N/A	Use of metformin, DPP-4i, GLP1 analogs and SGLT2i inhibitors (OR 0.58 [95% CI: 0.42–0.81]) was associated with lower odds of dementia after multible adjustments
Mui J. V. et al. (China) [23] (2021)	Longitudinal Study	To evaluate the effects of the two novel antidiabetic agents on cognitive dysfunction by comparing the rates of dementia between SGLT2I and DPP4I users.	51,460 patients	66.3 [58–76 IQR] years	not reported	472 days	SGLT2I use was associated with lower risks of dementia (hazard ratio HR 0.41, [95% CI 0.27–0.61]), Parkinson (HR 0.28 [95% CI 0.09–0.91]), all-cause (HR 0.84, [95% CI 0.77–0.91]), cardiovascular (HR 0.64, [95% CI 0.49–0.85]) and cerebrovascular (HR:0.36, [95% CI 0.30–0.43]) mortality
Mone P. et al. (Italy) [24] (2022)	Longitudinal Study	To assess cognitive and physical function in consecutive frail older adults with diabetes and HFpEF, comparing the effects of empagliflozin, metformin and insulin	52 empaglifozin users, 56 metformin users, 54 insulin users	80 [6.6 SD] years in the empaglifozin group, 80 [6.3 SD] years in the metformin group, 81.4 [5.5 SD] years in the insulin group	empaglifozin	1 month	The multivariable regression analysis showed the beneficial effects of empagliflozin (OR 3.61; [95% CI 1.57–8.32]) and no effect of metformin and insulin therapy.
Low S. et al. (Singapore) [25] (2022)	Longitudinal Study	To evaluate the possible association between SGLT2i and longitudinal changes in cognitive functions in patients with Type II Diabetes	138 patients with SGLT2i	60.6 [7.4 SD] years	not reported	4.6 years	The use of SGLT2i was associated with an increase in the Repeteable Battery for the Assessment of Neuropsychological Status (RBANS) total score in language (*p* = 0.019)
Proietti R. et al. (International dataset) [26] (2023)	Longitudinal Study	To investigate cardiovascular, cerebrovascular and cognitive outcomes of SGLT2i therapy in patients with atrial fibrillation (AF) and T2DM	89356 patients with AF	71.8 [11.3 SD] years in the control group; 66.6 [9.92 SD] years in the SGLT2i group	not reported	3 years	the risk of ischemic stroke/TIA was higher in patients not receiving SGLT2i (HR 1.12 [95% CI 1.01–1.24]) and for ICH (HR 1.57 [95% CI 1.25–1.99]) and incident dementia (HR 1.66 [95% CI 1.30–2.12])
Ding J et al. (China) [27] (2023)	Longitudinal Study	To investigate the correlation between long-term glycemic variability and cognitive function in middle-aged and elderly patients with type 2 diabetes mellitus (T2DM)	222 patients	63.1 (57.00–69.50 IQR) years in the MCI group; 60.7 (56.0–65.0 IQR) years in the MCI-free group	not reported	16.1 months	HbA1c SD, fasting glucose SD and smoking were risk factors for cognitive dysfunction, while eGFR, GLP-1RA and SGLT-2i usage had a protective effect.
Merzon E. et al. (Israel) [28] (2024)	Cross-sectional study	To assess the prevalence, clinical characteristics and healthcare utilization of patients with type 2 diabetes and previously undiagnosed cognitive impairment (who were identified as having a low MoCA score)	350 patients	73.8 [5.8 SD] years	not reported	N/A	Patients with MoCA < 19 had more diabetes-related complications, were less likely to be treated with GLP-1Ra, DPP-4i or SGLT2i and were more likely to receive insulin or sulfonylurea
